# Recent evidence on rates and factors influencing smoking behaviours after release from smoke-free prisons: a scoping review

**DOI:** 10.1108/IJOPH-10-2023-0064

**Published:** 2024-10-17

**Authors:** Ashley Brown, Clair Woods-Brown, Kathryn Angus, Nicola McMeekin, Kate Hunt, Evangelia Demou

**Affiliations:** Institute for Social Marketing and Health, Faculty of Health Sciences and Sport, University of Stirling, Stirling, UK; Health Economics and Health Technology Assessment (HEHTA), School of Health and Wellbeing, University of Glasgow, Glasgow, UK; Institute for Social Marketing and Health, Faculty of Health Sciences and Sport, University of Stirling, Stirling, UK; MRC/CSO Social and Public Health Sciences Unit, School of Health and Wellbeing, University of Glasgow, Glasgow, UK

**Keywords:** Prison, Smoking, Inequalities, Vaping, Cessation interventions, Marginalised populations, Smoke-free policy

## Abstract

**Purpose:**

Smoke-free prison policies have been introduced in some countries, in part to address very high levels of tobacco use in people in prison. However, relapse rates post-release remain high. This papers aims to improve understanding of post-release smoking and/or vaping behaviour is necessary to inform support for a priority population.

**Design/methodology/approach:**

The authors searched health, social science and criminal justice databases for studies about smoking/vaping behaviours among people released from smoke-free prisons. Studies were included if they reported primary data and were published between January 2017 and March 2024 in English; the population was adults/young people (16 yr+) imprisoned or formerly imprisoned, in prisons with comprehensive smoke-free policies; and at least one of the following was reported: pre-release intention to smoke, vape or remain abstinent post-release; smoking/vaping behaviour post-release and factors influencing smoking/vaping behaviour; attempts to quit again following post-release smoking/vaping relapse.

**Findings:**

Nine studies met our criteria. The evidence base is small and mainly from the USA or Australia. Evidence continues to suggest that most people resume smoking after leaving a smoke-free prison. No new interventions have been successful in reducing relapse rates. No studies report on vaping post-release, although two studies report on perceived factors affecting smoking relapse post-release from prisons allowing vaping.

**Research limitations/implications:**

Given very high rates of relapse, there remains a significant need to better understand what approaches are feasible and acceptable for reducing return to smoking post-release.

**Originality/value:**

This review updates the limited evidence on smoking behaviours after leaving a smoke-free prison.

## Introduction

Tobacco use continues to be one of the most serious public health threats internationally, accounting for more than 7.69 million (7.16–8.20) deaths and 200 million (185–241) disability-adjusted life-years in 2019, and is still the leading risk factor for death in men (GBD 2019 Tobacco Collaborators, 2021). Although smoking rates have fallen in many resource-rich countries, smoking prevalence remains high among “marginalised” groups (Potter *et al.*, 2021). A systematic review of smoking prevalence in prisons across 36 countries where smoking was/is still permitted reported that the rates of smoking were up to five times higher than in the general population in Europe, up to 6.4 times higher in the Americas and up to 16 times higher in Asia (Spaulding *et al.*, 2018). For example, in Scotland, in 2017, prior to the 2018 smoke-free prison policy, 68% of people in prison used tobacco compared with 18% of adults outside prison (Carnie *et al.*, 2017; SHeS, 2017) and levels of second-hand smoke (SHS) in prisons were comparable to those within a typical smoking home (Semple *et al.*, 2017). Thus, people who are or have been in prison are at greater risk of smoking-related health conditions than the general population (Binswanger *et al.*, 2014).

Several countries have introduced total smoke-free prison policies to tackle the high levels of smoking among people in prison, exposure to SHS and tobacco-related mortality and morbidity for tobacco users and non-users (Sweeting and Hunt, 2015). New Zealand was the first country to implement a comprehensive country-level smoke-free prison policy (Collinson *et al.*, 2012). Smoke-free prison policies have also been implemented in most jurisdictions in Australia (Office of The Inspector of Custodial Services, 2021) and in the USA (Binswanger *et al.*, 2014) and Canada (Collier, 2013). All prisons in England and Wales had become smoke-free by July 2018 (ASH, 2018). Scotland implemented its smoke-free prison policy in November 2018 (Hunt *et al.*, 2022). UK jurisdictions elected to allow vaping in smoke-free prisons, as have some USA prisons (Young-Wolff *et al.*, 2015).

Evidence shows environmental improvements following smoke-free prison policy. For example, measurements in Scottish prisons, during the week of smoke-free prison policy implementation, demonstrated an immediate impact (81% reduction in SHS) (Semple *et al.*, 2020); levels were further reduced six months later (Demou *et al.*, 2020). Other studies have reported reductions in SHS markers of 77% in North Carolina (Proescholdbell *et al.*, 2008), 50% in New Zealand (Collinson *et al.*, 2012) and 66% in four pilot prisons in England (Jayes *et al.*, 2019).

Some studies have reported evidence of health benefits of smoke-free prison policies. For example, a USA study reported a 9% reduction in smoking-related deaths, particularly from heart and lung disease, in prisons where a smoke-free policy had been in place for nine or more years. There was also a reduction in cancer-related deaths (Binswanger *et al.*, 2014). In England, a reduction in risk of a cardiovascular event across all ages groups has been evidenced (Perrett *et al.*, 2022). In Scotland, a time-series analysis of prison pharmacy data suggested health benefits evidenced by a 9% reduction in the rate of dispensing medication for smoking-related illnesses (Tweed *et al.*, 2021). Health economic analyses demonstrated cost-effectiveness of the Scottish smoke-free prison policy over the short- and long-term, but uncertainty remained in the long-term modelling due to the paucity of evidence internationally about smoking following release from smoke-free prisons (McMeekin *et al.*, 2022).

A systematic review of prison-based smoking cessation interventions concluded that “prisoners who experience a complete smoking ban typically resume smoking shortly after release” suggesting that, on their own, smoke-free policies are insufficient to maintain smoking abstinence after release (de Andrade and Kinner, 2017). Another systematic review identified 15 studies published to July 2017 with data on pre-release intentions around smoking, smoking relapse or abstinence post-release or quit attempts following post-release smoking relapse and noted that the evidence base was “small, almost exclusively US-based” and “methodologically weak” (Puljević and Segan, 2019). Their review highlighted “that more than 60% of participants relapsed to smoking following release from smokefree prisons” (p. 1015). Both of these reviews were conducted before e-cigarette use was permitted in smoke-free prisons in the UK, and neither considered whether the use of e-cigarettes in a smoke-free prison affects intention to or return to smoking rates post-release.

Spaulding *et al.* (2018) have estimated that in-prison policy programmes could potentially result in 2 million fewer “smokers” resettling in communities globally. However, high rates of relapse to smoking suggest that opportunities to improve the health of people who have been incarcerated in smoke-free prisons are being missed, particularly where the use of e-cigarettes is not permitted and the period of imprisonment extends beyond two to three months. Symptoms of nicotine withdrawal peak in the first week of abstinence, with some studies suggesting they return to baseline levels by two to four weeks, and others that they persist for several months (Hatsukami *et al.*, 2008). The health of people released from prison is complex due to co-occurring physical (Ismail *et al.*, 2021) and mental health conditions (Fazel and Seewald, 2018), entrenched and intergenerational social disadvantage (Enggist *et al.*, 2014) and persistent inequalities in the use and effects of tobacco smoking (Hiscock *et al.*, 2011). For example, recent Australian research reports that despite very high levels of mental illness and complex multimorbidity, continuity of care post-release is inadequate (Calais-Ferreira *et al.*, 2022) and people released from prison with mental health and substance use issues have a high risk of morbidity, mortality and reincarceration (Thomas *et al.*, 2022; Borschmann *et al.*, 2024).

With recent evidence of high rates of relapse to smoking post-release, up to date understanding of post-release smoking/vaping behaviour is necessary to increase understanding of appropriate support strategies. This scoping review builds on and expands earlier reviews (Puljević and Segan, 2019; de Andrade and Kinner, 2017) by updating evidence on relapse rates and factors influencing smoking behaviours post-release and explicitly searching for relevant research on e-cigarettes.

## Methods

The paper was informed by the Preferred Reporting Items for Systematic reviews and Meta-Analyses extension for Scoping Reviews (Tricco *et al.*, 2018) (Supplementary File 1).

### Search strategy

Relevant search terms were identified, informed by the previous systematic review and the research team’s expertise in tobacco use in prisons and used to construct a detailed search strategy for five databases according to the PICO model (Population, Intervention, Context, Outcome) (Supplementary File 2) to update and broaden earlier reviews (de Andrade and Kinner, 2017; Puljević and Segan, 2019). We tested the search terms to ensure they identified papers in the previous review. We searched Web of Science Citation Indices, PsycInfo (EBSCOHost), Criminal Justice Abstracts (EBSCOHost) and Medline (Ovid) databases, and the Bielefeld Academic Search Engine (BASE), on 6 April 2022; the search was updated on 5 March 2024. The database search was limited to English language records, and included journal articles, dissertations and grey literature, such as third-sector reports and government documents. To identify further relevant studies, we used backward snowballing and forward plus emailed autoalerts of new records from the academic databases.

### Inclusion and exclusion criteria

Our examination of studies included vaping because some jurisdictions (e.g. Scotland and England and Wales, some prisons in the USA) opted to allow use of e-cigarettes in smoke-free prisons. Our earlier work suggests that uptake has been high in Scotland (Brown *et al.*, 2021; Brown *et al.*, 2020; Brown *et al.*, 2019; Hunt *et al.*, 2022). In England, it has also been reported that the majority of “smokers” living in closed prison environments vape to manage without tobacco (Jayes *et al.*, 2023).

Studies were included if they reported primary data and were published between January 2017 and March 2024; they were published in English; the population was adults/young people (16 yr+) imprisoned or formerly imprisoned, in prisons with comprehensive smoke-free policies; and the study reported at least one of the following:

- pre-release intention to smoke or vape or remain abstinent post-release;
- smoking/vaping behaviour post-release and factors influencing smoking/vaping behaviour; and
- attempts to quit again following post-release smoking/vaping relapse.

Studies were excluded if they reported on smoking or vaping behaviours during imprisonment only.

### Data charting

A data extraction table to record characteristics of the studies and the outcomes of interest for our synthesis was co-created by two reviewers, tested using pilot studies by three reviewers and the final data extraction was sense-checked by a fourth with reference to all included studies.

## Results

### Search results

**Figure 1:**
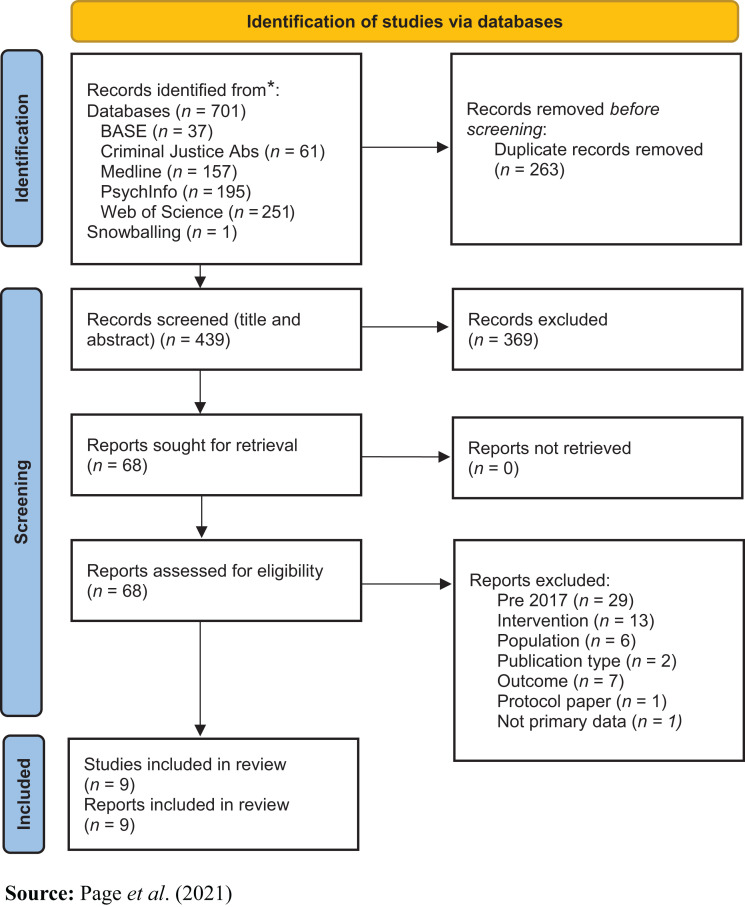
PRISMA flow diagram of identification of studies

The database search identified 702 records that were imported into a reference manager (EndNoteX9). After eliminating duplicates, 439 records remained and were imported into an online systematic review tool (www.rayyan.ai). Titles and abstracts were independently screened for eligibility by two reviewers, with any disagreements resolved by a third. A total 369 were excluded and 68 were retrieved for full text screening. Studies pre-2017 included in either of the previous reviews (de Andrade and Kinner, 2017; Puljević and Segan, 2019) and the reviews themselves were then excluded (*n* = 29). Further studies were excluded on the basis of intervention (*n* = 13), publication type (*n* = 2), population (*n* = 6) or outcome (*n* = 7). A protocol paper and a review paper were also excluded. Thus, nine studies (all peer-reviewed journal articles reporting primary data) met the inclusion criteria after full-text review. Figure 1 details the search and retrieval process (Page *et al.*, 2021).

### Overview of included studies

Table 1 describes the characteristics of nine included studies. Four of the studies reported studies in Australia (Jin *et al.*, 2021; Albany *et al.*, 2020; Puljević *et al.*, 2018; Puljević *et al.*, 2019) (in states where smoke-free policies were in place in prisons), three were based on studies conducted in the USA (Ives *et al.*, 2022; Martin *et al.*, 2022; Winkelman *et al.*, 2021) (in states with smoke-free prisons) and two were from the UK. The two qualitative studies based in the UK made explicit reference to the permitted use of vaping in smoke-free prisons. One reports on interviews with people working in three all male prisons or people tasked with implementing smoke-free prison policies within Public Health England or the headquarters for the prison services, which included questions on the management of nicotine addiction in prison and after release (Jayes *et al.*, 2023). The other also mainly focused on the implementation of smoke-free policy in prisons but included some data on which factors were perceived to hinder or facilitate remaining smoke-free on release from interviews with prison staff and 18 men and five women in custody (Brown *et al.*, 2022).

All nine studies reported on adults, seven measured outcomes for mixed sex, although predominantly male samples (Albany *et al.*, 2020; Jin *et al.*, 2021; Martin *et al.*, 2022; Puljević *et al.*, 2018; Winkelman *et al.*, 2021; Puljević *et al.*, 2019; Brown *et al.*, 2022); one study only included men (Ives *et al.*, 2022). Three studies used qualitative methods only, reporting on data from semi-structured interviews (Brown *et al.*, 2022; Jayes *et al.*, 2023; Puljević *et al.*, 2019) and three reported on cross-sectional surveys (Albany *et al.*, 2020; Ives *et al.*, 2022; Puljević *et al.*, 2018).

Three studies reported data from evaluations of interventions aimed at reducing return to smoking post-release. All three studies involved provision of (relapse prevention) behavioural support for smoking cessation (Jin *et al.*, 2021; Martin *et al.*, 2022; Winkelman *et al.*, 2021). In one study, the intervention group received behavioural support (motivational interviewing) pre-release (Jin *et al.*, 2021). In another study, the intervention involved provision of smoking cessation counselling in-person for one hour pre-release, and up to four telephone sessions in the three weeks post-release (Winkelman *et al.*, 2021). Participants were also provided with nicotine lozenges in addition to behavioural support (Winkelman *et al.*, 2021). The third intervention (known as the Working Inside for Smoking Elimination [WISE] intervention) involved receipt of six motivational interviewing or cognitive behaviour therapy sessions in the ∼6 weeks pre-release, and brief telephone sessions around one-day and one-week post-release (Martin *et al.*, 2022). More details about the broader WISE study from which Martin *et al.*’s subsample derives are reported elsewhere (Clarke *et al.*, 2013).

This scoping review considered four smoking outcomes:

1. pre-release intentions to smoke or vape or remain abstinent following release;
2. the prevalence of smoking or vaping after release;
3. quit attempts following post-release relapse; and
4. factors facilitating or hindering staying smoke-free or reducing levels of tobacco consumption post-release including the use of e-cigarettes.

Given the heterogeneity in the focus and methodologies used in the included studies, it is only possible provide a narrative summary of outcomes.

## Reported outcomes of included studies

Only two of the nine studies included in our review reported on any aspect of vaping. We found no studies reporting on intention to vape post-release (as a way to remain smoke-free) or on prevalence of vaping post-release. Hence, there is currently no evidence internationally on whether, or how, vaping while in a smoke-free prison or following release affects intentions to remain smoke-free, or rates of relapse to smoking, after release from smoke-free prisons.

Similarly, none of the studies reported on quit attempts following return to smoking after leaving a smoke-free prison.

### Pre-release intentions to smoke or remain abstinent following release from a smoke-free prison

Six studies included information on participants’ pre-release intentions/expectations to smoke/remain abstinent on release (Ives *et al.*, 2022; Jin *et al.*, 2021; Martin *et al.*, 2022; Puljević *et al.*, 2019; Puljević *et al.*, 2018; Winkelman *et al.*, 2021). The percentage who expressed pre-release intentions/expectations to smoke/remain abstinent varied across studies, with four studies reporting that at least one in three participants expected, intended or hoped to remain smoke-free. Only two studies, with different designs, recorded pre-release intentions to remain abstinent after release prospectively, while participants were still in prison. The first, a survey conducted across a release preparation program in the USA within 20 weeks of release, reported 33% of respondents, in the 2020 survey wave, selected “definitely not” when asked whether they expected to resume smoking on release (Ives *et al.*, 2022). The second, an RCT of a motivational interview intervention conducted with 557 participants within four to six weeks of release from an Australian prison reported that at baseline 51% of participants who were allocated to the intervention group “hoped to remain smokefree after release” compared to 43% of the control group (Jin *et al.*, 2021). However, one inclusion criterion assessed for participation in the trial was that potential participants “express an interest in remaining abstinent after release …” (p. 464).

Four studies measured pre-release intentions to smoke/remain abstinent retrospectively. One, a cross-sectional survey of 114 participants released from smoke-free prisons in Queensland, Australia (Puljević *et al.*, 2018), reported that 66% of participants expressed an intention to remain smoke-free. In a qualitative study, which subsampled from participants in this survey who had relapsed to smoking, 12 of the 21 participants were reported to have expressed a “pre-release intention to remain abstinent after release” (Puljević *et al.*, 2019). A secondary analysis of a subsample (*n* = 190) from the WISE project reported that 52% planned to return to smoking on release (Martin *et al.*, 2022).

### Smoking after release

Five studies included information on the prevalence of smoking after release from a smoke-free prison (Albany *et al.*, 2020; Jin *et al.*, 2021; Martin *et al.*, 2022; Puljević *et al.*, 2018; Winkelman *et al.*, 2021). Timepoints at which smoking prevalence data were collected, and outcome measures varied across the studies, preventing detailed comparisons being made across studies. All studies found that at least ∼65% returned to smoking. Among 424 participants followed up (for an average of ∼145 days) in an Australian RCT of a motivational interviewing intervention, most participants had returned to smoking according to several measures (Jin *et al.*, 2021). Continuous smoking absence was self-reported by 16% (*n* = 31) in the intervention group and 12% (*n* = 27) of the control group and seven-day point prevalence for self-reported abstinence was 31% in both groups. Rates of abstinence were lower for the primary outcome (continuous absence [not having smoked more than five cigarettes during follow-up period] confirmed by exhaled carbon monoxide); after multiple imputations to account for missing data, continuous abstinence was estimated to be 9% in the intervention group and 7% in the control group. Cumulative self-reported relapse at three months was 87% for the intervention group and 92% for the control group; whereas 62% and 74%, respectively, reported relapse on the day of release. The median number of cigarettes smoked daily was five and six, respectively (Jin *et al.*, 2021). Another Australian study reported that 72% of self-identifying “smokers” (*n* = 114) released from prison in Queensland relapsed to smoking within 24 hours and 94% within two months of release (Puljević *et al.*, 2018) (although 62% of those who relapsed to smoking post release reported smoking fewer cigarettes post-release than pre-incarceration), whereas a third Australian study reported that 86% of people re-entering prison after being in a smoke-free prison in the past 12 months were daily “smokers” (Albany *et al.*, 2020).

Of the 190 participants from the WISE study in the USA, 50% relapsed to smoking within 24 hours and 65% within seven days (Martin *et al.*, 2022). The adjusted biologically verified seven-day point prevalence abstinence (primary outcome) in a pilot RCT in the USA was 12% in a group who received 1 h of smoking cessation counselling in jail plus a supply of NRT on release, compared to 11% for the group who received 30 min of general health education only; equivalent figures at 12 weeks were 11% and 14%, respectively (Winkelman *et al.*, 2021).

One study suggested that there may be a reduction in numbers of cigarettes smoked (Puljević *et al.*, 2018), comparing the number of cigarettes smoked before and after time spent in a smoke-free prison. Two studies (Jin *et al.*, 2021; Winkelman *et al.*, 2021) found a potential reduction in the number of cigarettes smoked post-release following participation in a relapse prevention intervention.

### Factors facilitating or hindering staying smoke-free or reducing levels of tobacco consumption post-release

Four studies using quantitative methods (Albany *et al.*, 2020; Martin *et al.*, 2022; Puljević *et al.*, 2018; Ives *et al.*, 2022) and three studies using qualitative methods (Puljević *et al.*, 2019; Brown *et al.*, 2022; Jayes *et al.*, 2023) described factors that hindered remaining abstinent from smoking. Factors identified in studies using quantitative methods included indigenous status (Albany *et al.*, 2020) and use of drugs (“any drug use” in the past four weeks (Albany *et al.*, 2020), “injectable drug use since release” (Puljević *et al.*, 2018) as predictors for daily smoking; sex (women at higher risk of smoking in first hour, day and week of release) (Martin *et al.*, 2022) and planning to smoke following release (Martin *et al.*, 2022). A nine-year survey of people in prison identified factors related to individual beliefs and expectations about smoking that may hinder abstinence; these included no longer viewing themselves as addicted to nicotine and believing it would be “fairly easy” not to return to smoking post-release (Ives *et al.*, 2022). Another study (Martin *et al.*, 2022) that examined the antecedents of return to smoking post-release identified several circumstances commonly associated with smoking after someone leaves prison: being with other people who smoke or with friends and family; being given tobacco by someone else; feeling positive emotions (e.g., being happy or excited); and using drugs or alcohol.

A qualitative study conducted in Australia identified pre-release intention, normalisation of smoking in home or social environments, perception of smoking as a symbolic act of freedom or resistance or as providing a means to relieve stress associated with resettlement or coping with craving for illicit substances, as barriers to continued abstinence after leaving a smoke-free prison (Puljević *et al.*, 2019). Some similar factors were also cited in two qualitative studies that explored *anticipated* barriers to remaining smoke-free post-release (Brown *et al.*, 2022; Jayes *et al.*, 2023). In addition, staff associated with the implementation of smoke-free prisons in England felt that an additional potential reason for smoking relapse post-release was high levels of nicotine use in the prison population due to vaping (Jayes *et al.*, 2023). Staff described the high prevalence of return to smoking when people moved from smoke-free closed prisons to the open estate (that were not smoke-free) (Jayes *et al.*, 2023). They suggested that the stress of moving between closed and open prison conditions, the provision of a “smoking pack” and the fact that people were once again surrounded by people smoking, made it more difficult for people to remain smoke-free. Jayes *et al.* noted that around 30% of people in the open prison context were using e-cigarettes, but “most of these” were dual users of cigarettes and e-cigarettes.

The most cited facilitators mentioned by 72 survey participants (Martin *et al.*, 2022) who managed to remain smoking abstinent post-release were: not having cravings to smoke, being with family, lack of finances, engaging in alternative activities, having willpower and thinking about the health benefits of not returning to smoking. Living with a partner, expressing support for smoke-free prison policy, pre-release intention to remain abstinent and future intention to quit smoking were associated with reduced daily smoking in another survey (Puljević *et al.*, 2018). Participants in the Australian qualitative study of 21 people who had managed to remain abstinent for a time post-release (Puljević *et al.*, 2019) mentioned awareness of health and financial benefits of not smoking, diversionary activities, social support from family and peers and intrinsic motivation. People in prison in Scotland thought the experience of health and financial benefits of not smoking while in prison may increase motivations to give up in the longer term, and that use of smoking alternatives during the transition period may help some people to avoid relapse (Brown *et al.*, 2022).

One study (Jin *et al.*, 2021) suggested that a single session of a motivational interviewing intervention delivered prior to release may potentially delay relapse to smoking post-release.

## Discussion

This scoping review builds on the systematic review by Puljević and Segan (2019) of studies reporting on factors influencing smoking following release from smoke-free prisons. Their review identified 15 eligible studies and concluded that “the evidence base in this area is small […] [and] mostly methodologically weak” (p. 1011) and dominated by studies from the USA. Our review (of studies published from 2017 to March 2024) demonstrates that the evidence base remains “sparse” and identified only an additional nine studies, of varying methodological design and quality. Most were conducted in the USA or Australia. A systematic review (of studies published from January 2000 to February 2022), which examined the impact of smoking “bans” and other smoking cessation interventions in smoke-free prisons, mental health and substance use treatment settings, similarly reported a dearth of evidence on post-release/post-discharge smoking behaviours (Sourry *et al.*, 2022).

Consistent with existing evidence, our review found that most people return to smoking after leaving a smoke-free prison, despite many people hoping or intending to remain smoke-free. This is a missed opportunity to improve the health of a priority group and to reduce health inequalities.

None of the new interventions (first published after 2017) reported in the studies included in this review have been successful in preventing high rates of relapse to smoking, often within 24 h, following release from a smoke-free prison. To our knowledge, the WISE intervention remains the only intervention, which has shown effectiveness in increasing smoking abstinence post-release (Clarke *et al.*, 2013). Nonetheless, one study in our review reports that a single session of motivational interviewing delivered pre-release (Jin *et al.*, 2021) may have the potential to delay return to smoking. This finding could be exploited in the future through the inclusion of greater support, including in the post-release period, which may help to maintain smoking abstinence for a longer period of time and/or facilitate further quit attempts in the event of a smoking lapse. Our review also found evidence that smoking levels may reduce following time spent in a smoke-free prison (Puljević *et al.*, 2018), and following participation in a pre-release motivational interviewing intervention (Jin *et al.*, 2021) or a pre–post intervention combining behavioural support and NRT (Winkelman *et al.*, 2021). As noted elsewhere (Winkelman *et al.*, 2021), the significance of results suggesting that some interventions may potentially reduce the amount smoked post-release is less clear given uncertainties about the implications for health and long-term smoking abstinence (Chang *et al.*, 2020; Lindson *et al.*, 2019). Taken together, evidence from this review and from previous reviews by Puljević and Segan (2019) and Sourry *et al.* (2022) highlight the potential of multi-component interventions that are delivered both before and after release to change smoking behaviour.

Puljević and Segan (2019) noted the need for evidence on the potential for NRT to help reduce return to smoking post-release. We identified one intervention study in which participants received a supply of nicotine lozenges upon release from prison in combination with behaviour support. Although the intervention did not increase smoking abstinence, participants were positive about the behavioural support provided and suggested that the provision of NRT could be expanded to in prison use, particularly in the lead up to release. Given that a systematic review of effective behaviour change techniques for preventing relapse following a stay in a smoke-free setting identified pharmacological support as effective in reducing return to smoking (Shoesmith *et al.*, 2021), further research on the use of NRT during and following time spent in a smoke-free prison would be beneficial. Specifically, it would be helpful to understand more about the optimum timing of providing NRT, the most feasible and acceptable mechanisms for giving people access to NRT and how to maximise uptake and adherence.

Crucially, our review demonstrated a complete lack of evidence worldwide on rates of relapse to tobacco use following release from smoke-free prisons where the sale and use of e-cigarettes has been allowed (as has been the case in England and Wales and in Scotland over the past five years, and in some prisons in the USA). Although evidence shows that e-cigarettes can help people to cope with smoke-free rules while living in prison (Brown *et al.*, 2020; O'Donnell *et al.*, 2022), research is required to understand whether and for whom vaping is a risk or protective factor for smoking relapse after leaving a smoke-free setting. To date, evidence from the general population is mixed. For example, a representative sample of USA recent former “smokers” did not find an association between daily vaping and remaining abstinent. However, it was found that those using e-cigarettes appeared more likely to quit again following a relapse and to be abstinent for three months at follow-up (Pierce *et al.*, 2021).

Given that vaping is not risk-free (Glantz *et al.*, 2024; McNeill *et al.*, 2022), it will be important to also assess levels and patterns of dual use of tobacco and e-cigarettes among prison leavers and to tailor support to try to encourage complete tobacco cessation in this group.

This review demonstrates that more research is needed on the most feasible and effective means to support people leaving smoke-free prisons to remain smoke-free. It strengthens understanding of factors potentially facilitating or hindering smoking abstinence post-release and which may help optimise support with smoking behaviour among people leaving smoke-free prisons. Firstly, strengthening intentions and plans to remain smoke-free post-release should continue to be an important goal for interventions (Martin *et al.*, 2022; Puljević *et al.*, 2019; Puljević *et al.*, 2018). Secondly, interventions could target incomplete understandings that people in prison may have about the nature of smoking addiction and about the (very high) risks of returning to smoking post-release (Ives *et al.*, 2022). Thirdly, interventions could support people to prepare coping strategies for when they are likely to be faced with smoking triggers almost immediately after leaving prison (e.g. feeling buoyant about release, returning to the company of people who smoke and being offered tobacco) (Martin *et al.*, 2022). Developing coping strategies for ongoing social and environmental smoking triggers associated with resettlement from prison (Puljević *et al.*, 2019) is also likely to be beneficial and could be addressed as part of providing wraparound services for people exiting prison. Co-use of tobacco and alcohol/illicit drugs among people leaving prison (Albany *et al.*, 2020; Martin *et al.*, 2022; Puljević *et al.*, 2019) continues to be an important risk factor for smoking behaviour interventions. Interventions need to be cognisant of polysubstance use to improve outcomes (McKelvey *et al.*, 2017). Finally, family-based interventions could be developed given continued evidence of the positive/negative influence which social networks have on smoking behaviours (Puljević *et al.*, 2019; Martin *et al.*, 2022).

This review suggests several areas to potentially target to try to address very high rates of smoking relapse following release from smoke-free prison. However, despite there being clear public health benefits to people remaining smoke-free post-release, there remains uncertainty about how best to intervene in this population due to the limited evidence-base. Further research to better understand what approaches are both feasible and effective for maintaining smoking abstinence following release from prison is needed. This could include studies to support tailoring of existing interventions for smoking cessation/abstinence to reflect the complex needs of people in prison, the challenges they face as they return to the community and to overcome constraints for delivering health promotion activities in specific prison settings. Studies to help maximise the potential of novel interventions that look promising would also be beneficial. Difficulties in implementing and researching new health initiatives in prisons may help to explain the current limited evidence available to help those seeking to maximise the health gains of smoke-free prison and other similar settings (Sourry *et al.*, 2022). Such difficulties include competing priorities, considerable constraints on access and resources and features of the setting or the population which can make randomisation and follow-up post-release challenging and costly (Sourry *et al.*, 2022).

A strength of our review is that we searched databases that cover health, justice and criminology. We have updated previous reviews at a time when further jurisdictions (England, Wales and Scotland) have introduced smoke-free prison policies; it is noteworthy that these UK jurisdictions elected to allow vaping in smoke-free prisons. However, our review is limited by the sparse evidence base, which limits our ability to draw firm conclusions. As in the earlier systematic review (Puljević and Segan, 2019), our searches were restricted to studies published in English and the evidence base remains dominated by studies from Australia and the USA. A notable finding is that there is currently no evidence internationally on whether, or how, vaping whilst in a smoke-free prison or following release affects intentions to remain smoke-free, or rates of relapse to smoking, after release from smoke-free prisons.

## Conclusion

All evidence to date shows that relapse rates post-release remain high, thus diminishing the public health potential of smoke-free prison policy and limiting the extent to which such policies may help to reduce health inequalities attributable to smoking. This highlights a significant need to continue to try to develop the evidence base to support stakeholders to better equip those leaving prison with the skills, motivation and support to remain smoke-free post-release.

## Acknowledgment

This study is funded by the NIHR [PHR Project: NIHR 131613]. ED further acknowledges funding from the Medical Research Council (MC_UU_00022/2) and the Chief Scientist Office (SPHSU17). For the purpose of open access, the author(s) has applied a Creative Commons Attribution (CC BY) licence to any author-accepted manuscript version arising from this submission. The views expressed are those of the author(s) and not necessarily those of the NIHR or the Department of Health and Social Care.

The authors would like to thank Ms Valerie Wells (SPHSU Information Scientist) for her assistance in running the database searches.

## Supplementary Material





## Figures and Tables

**Table 1:** Characteristics of the included [studies/publications] and smoking outcomes measured

Authors	Country	Sample (size and gender)	Study design	Outcomes included	Timepoints of measures
Albany *et al.* (2020)	Australia	389 prison entrants (18+) participated (85% male). 152/389 were imprisoned in previous 12 months in jurisdictions with total smoking ban [“exposed”]; “nonexposed” were in jurisdictions without total smoking bans/not in prison in previous 12 months; missing data on exposure for *n* = 23	Sample derived from 2016 wave of National Prisoner Entrants’ Blood Bourne Virus Survey of prison entrants at 19 sites across six Australian jurisdictions. Cross-sectional survey included four questions on tobacco use	Self-reported daily tobacco smoking immediately prior to reimprisonment	Survey conducted immediately after routine health and welfare assessments as individuals enter prison
Brown *et al.* (2022)	Scotland	99 prison staff; 23 people in custody (18 men, 5 women)	Focus group discussions (FGDs) with staff (one group in each of 14 closed prisons, 95 staff in total), one-to-one interviews (23 people in custody)	Topics covered included: opinions of smoke-free prison policy; perspectives on living/working in a smoke-free prison; compliance and enforcement of smoke-free prison policy; lessons learned	FGDs and interviews conducted 6–9 months after implementation of smoke-free prison policy in all Scottish prisons in November 2018
Ives *et al.* (2022)	USA	5,289 men admitted to a release preparation programme in prisons admitting people with substance use disorders	5- to 10-min survey completed by admissions between 2012 and 2020. Questions were modified periodically so answers for some questions only available for subset	Pre-release intention to remain abstinent	∼20 weeks pre-release
JAYES *et al.* (2023)	England	28 stakeholders with key strategic and/or operational roles in delivering smoke-free prison policy. Purposive sampling to identify those involved in smoke-free prison policy within HMPPS Headquarters and regionally and with HMPPS and health-care staff at three male prisons	Semi-structured in-person/ telephone one-to-one interviews	Stakeholder views on the implementation and delivery of complete and partial smoke-free policies, specifically in relation to the management of nicotine addiction whilst in prisons and after release	Conducted August-November 2019, i.e. four years after open estate introduced partial smoke-free policy and ∼4 years after last closed prison implemented a complete smoke-free policy
Jin *et al.* (2021)	Australia	557 individuals expected to be within four to six weeks of release randomised to either motivational interview intervention (*n* = 226; 93% male) or control (*n* = 291, 91% male) group. 424 were followed up post-release	RCT of smoking, nutrition, alcohol and physical (SNAP) inactivity intervention Self-report survey Smoking abstinence verified by exhaled carbon monoxide test	Pre-release intention to remain abstinent; self-reported continuous smoking abstinence and seven-day point prevalence abstinence, verified by exhaled carbon monoxide test for a subsample, (also self-reported number of cigarettes after release)	Four to six weeks pre-release Three months post-release
Martin *et al.* (2022) (WISE)	USA	190 participants (67% male) eligible for participation if within eight weeks of release	RCT of working inside for Smoking Elimination (WISE) intervention; computer-assisted survey and telephone follow-up	Pre-release intention to remain smoke-free post release. Self-reported smoking status post-release, time to relapse post-release. Antecedents of relapse	Eight weeks pre-release, 24-h and seven-days post-release
Puljević *et al.* (2018)	Australia	114 (former) smokers released from smoke-free prison in the previous two months (86% male)	Cross-sectional survey	Pre-release intention to remain abstinent; time to relapse post-release; rate of self-reported smoking post release	Within two-months post-release
Puljević *et al.* (2019)	Australia	21 people who had relapsed to smoking following release (76% male); subsample from Puljević *et al.* (2018)	Semi-structured interviews	Barriers and facilitators to maintaining smoke-free after release	Within two months of release
Winkelman *et al.* (2021)	USA	46 [former] smokers expected to be within 90 days of release and to have regular telephone access post-release (91% male)	Pilot RCT – counselling and NRT vs brief health education. Self-report baseline and follow-up survey	Self-reported smoking status post-release verified by exhaled carbon monoxide test	Baseline measures (pre-release), and 1, 3 and 12 weeks post-release

**Source:** Table by authors

## Supplementary material

The supplementary material for this article can be found online.
