# Supplementary material for: Recent evidence on rates and factors influencing smoking behaviours after release from smoke-free prisons: : a scoping review
Source: Int J Prison Health (2024). 2024 Oct 17;20(4):450–65. doi: 10.1108/IJOPH-10-2023-0064 (PMC11616588; doi:10.1108/IJOPH-10-2023-0064)
Supplement: Supplementary file 2 [file intjprisonhealth-20-0450-s002.docx]

Suppplementary file 2

Table S1: Example of Search Terms Used in Database Searches (table by author)

| **PICO heading** | **Search terms used to construct search strategies in each database** |
| --- | --- |
| **Population/Setting: Prison** | carceral*, convict*, correctional, corrections, custodial, custody, detain*, detention, felon*, gaol*, imprison*, incarcerat*, inmate*, jail*, offend*, penal, penitentiar*, prison*, remand* |
| **Intervention/Context:**  **Release from Smokefree Prison** | community supervis*, ex-convict*, ex-felon*, ex-inmate*, ex-offender*, ex-prisoner*, former*, freed, leav*, left, liberat*, parole*, postrelease, post-release, probation*, reenter*, re-enter*, reentries, re-entries, reentry, re-entry, reintegrat*, re-integrat*, releas* |
| **Outcome:**  **Tobacco/Vaping abstinence, intentions, behaviours** | cig*like*, cig-a-like*, cigar*, e-cig*, e-liquid, nicotine, non-nicotine, nonnicotine, *smoking, smok*, tobacco*, vape*, vaping, vaporiser/vaporizer |

Table S2: Example of the Full Electronic Search Strategy for APA PsycInfo, Including Limits Used.

| **Database: APA PsycInfo**  **Interface - EBSCOhost Research Databases**  Searched: 6^th^ April 2022  Search Screen - Advanced Search. ‘Apply equivalent subjects’ deselected. Search modes - Boolean/Phrase. | |
| --- | --- |
| S1 | TI (carceral* OR convict* OR correctional OR corrections OR custodial OR custody OR detain* OR detention OR felon* OR gaol* OR imprison* OR incarcerat* OR inmate* OR jail* OR offend* OR penal OR penitentiar* OR prison* OR remand*) OR AB (carceral* OR convict* OR correctional OR corrections OR custodial OR custody OR detain* OR detention OR felon* OR gaol* OR imprison* OR incarcerat* OR inmate* OR jail* OR offend* OR penal OR penitentiar* OR prison* OR remand*) |
| S2 | DE "Correctional Institutions" OR DE "Criminal Offenders" OR DE "Female Criminal Offenders" OR DE "Incarceration" OR DE "Legal Detention" OR DE "Male Criminal Offenders" OR DE "Prisoners" OR DE "Prisons" |
| S3 | S1 OR S2 |
| S4 | TI ((community N2 supervis*) OR exconvict* OR ex-convict* OR exfelon* OR ex-felon* OR exinmate* OR ex-inmate* OR exoffender* OR ex-offender* OR exprisoner* OR ex-prisoner* OR former* OR freed OR leav* OR left OR liberat* OR parole* OR postrelease OR post-release OR probation* OR reenter* OR re-enter* OR reentries OR re-entries OR reentry OR re-entry OR reintegrat* OR re-integrat* OR releas*) OR AB ((community N2 supervis*) OR exconvict* OR ex-convict* OR exfelon* OR ex-felon* OR exinmate* OR ex-inmate* OR exoffender* OR ex-offender* OR exprisoner* OR ex-prisoner* OR former* OR freed OR leav* OR left OR liberat* OR parole* OR postrelease OR post-release OR probation* OR reenter* OR re-enter* OR reentries OR re-entries OR reentry OR re-entry OR reintegrat* OR re-integrat* OR releas*) |
| S5 | DE "Criminal Rehabilitation" OR DE "Institutional Release" OR DE "Parole" OR DE "Probation" OR DE "Reintegration" |
| S6 | S4 OR S5 |
| S7 | TI (antismok* OR anti-smok* OR cig*like* OR cig-a-like* OR cigar* OR ecig* OR e-cig* OR eliquid* OR e-liquid* OR nicotine OR non-nicotine OR nonnicotine OR smok* OR tobacco* OR vape* OR vaping OR vapori?er*) OR AB (antismok* OR anti-smok* OR cig*like* OR cig-a-like* OR cigar* OR ecig* OR e-cig* OR eliquid* OR e-liquid* OR nicotine OR non-nicotine OR nonnicotine OR smok* OR tobacco* OR vape* OR vaping OR vapori?er*) |
| S8 | DE "Electronic Cigarettes" OR DE "Nicotine Withdrawal" OR DE "Nicotine" OR DE "Smoking Cessation" OR DE "Tobacco Smoking" OR DE "Tobacco Use Disorder" OR DE "Vaping" |
| S9 | S7 OR S8 |
| S10 | S3 AND S6 AND S9 |
| S11 | S10 Limiters - Language: English |
| S12 | S11 Limiters - Published Date: 20040101- |
